# Thermosensitive Nanocomposite Hydrogels for Intravitreal Delivery of Cefuroxime

**DOI:** 10.3390/nano9101461

**Published:** 2019-10-15

**Authors:** Simona Sapino, Elena Peira, Daniela Chirio, Giulia Chindamo, Stefano Guglielmo, Simonetta Oliaro-Bosso, Raffaella Barbero, Cristina Vercelli, Giovanni Re, Valentina Brunella, Chiara Riedo, Antonio Maria Fea, Marina Gallarate

**Affiliations:** 1 Department of Drug Science and Technology, University of Turin, 10125 Turin, Italy; elena.peira@unito.it (E.P.); giulia.chindamo@unito.it (G.C.); stefano.guglielmo@unito.it (S.G.); simona.oliaro@unito.it (S.O.-B.); marina.gallarate@unito.it (M.G.); 2 SC of Serology, Istituto Zooprofilattico Sperimentale Piemonte Liguria e Valle d’Aosta, 10154 Turin, Italy; raffaella.barbero@izsto.it; 3 Department of Veterinary Sciences of Turin, University of Turin, 10095 Turin, Italy; cristina.vercelli@unito.it (C.V.); giovanni.re@unito.it (G.R.); 4 Department of Chemistry, University of Turin, 10125 Turin, Italy; valentina.brunella@unito.it (V.B.); chiara.riedo@unito.it (C.R.); 5 Department of Surgical Sciences, University of Turin, 10126 Turin, Italy; antoniomaria.fea@unito.it

**Keywords:** cefuroxime, endophthalmitis, nanocomposite thermosensitive hydrogels, vitreous humor, ocular flow cell

## Abstract

Endophthalmitis is a rare, but serious, intravitreal inflammatory disorder that can arise after cataract surgery. The intracameral injection of 1 mg cefuroxime (CEF) followed by three-times daily antibiotic topical administration for a week is generally recognized as the routine method of prophylaxis after cataract surgery. This procedure is controversial because of both the low efficacy and the low adherence to therapy by elderly patients. A unique slow release antibiotic intravitreal injection could solve these problems. The objective of the present study was to design ophthalmic nanocomposite delivery systems based on in situ gelling formulations that undergo sol-to-gel transition upon change in temperature to prolong the effect of CEF. Oil in water (O/W) microemulsion (µE) and solid lipid nanoparticles (SLN), obtained with an innovative formulation technology called *cold microemulsion dilution*, were evaluated as ocular drug delivery systems for CEF. Drug entrapment efficiency up to 80% was possible by esterifying CEF with 1-dodecanol to obtain dodecyl-CEF (dCEF). Both dCEF-loaded SLN and µE were then added with Pluronic®F127 (20% *w*/*v*) to obtain a nanocomposite hydrogel-based long acting system. The prepared thermosensitive formulations were evaluated for their physical appearance, drug content, gelation temperature, injectability and rheological properties, in vitro release studies and stability studies. Moreover, cell proliferation assays on human retinal pigment epithelial ARPE-19 cells were performed to evaluate the influence of this innovative system on the cellular viability. In addition, minimal inhibitory concentration (MIC) was assessed for both CEF and dCEF, revealing the need of dCEF hydrolysis for the antimicrobial activity. Although further experimental investigations are required, the physico-chemical characterization of the nanocomposite hydrogels and the preliminary in vitro release studies highlighted the potential of these systems for the sustained release of CEF.

## 1. Introduction

Posterior segment of the eye is the portion of the eye that includes the anterior hyaloid membrane and all of the optical structures behind it: the vitreous humor, retina, choroid and optic nerve. It extends from the back surface of the lens to the retina and contains a gel-like fluid called the vitreous humor [1].

Several diseases and degeneration phenomena can affect the posterior segment, among which diabetic retinopathy, uveitis, retinitis, glaucoma and age-related macular degeneration. They are devastating, since in most cases these pathologies are causes of irreversible blindness [2,3]. Moreover, this part of the eye may encounter complications resulting from intraocular surgeries. Conventional topical eye drop administration is inadequate to treat the disorders affecting the posterior segment since it cannot reach the back section of the eye due to the presence of several physiological barriers [4,5]. 

Recently, new drugs for the medication of the posterior ocular segment, such as ranibizumab, bevacizumab and the antibody fragment aflibercept, have been proposed, and most of them are delivered by repeated intravitreal injections. However, the repeated and often needed long-term injections may cause complications, such as vitreous haemorrhage, retinal detachment, or endophthalmitis. Thus, the discovery of effective methods for posterior segment therapies is one of the greatest drug delivery challenge: effective, safe and comfortable methods of drug delivery are needed. Innovative methods include polymeric-controlled release injections and implants, micro- and nanoparticulates, iontophoresis, etc. [6,7].

In the recent decades, various nanoscale delivery systems have been introduced with the aim to improve solubility, stability and bioavailability of poorly absorbed drugs. Among all, solid lipid nanoparticles (SLN) represent an interesting approach for eye drug delivery as they can improve the drug bioavailability. Many research articles report SLN as an interesting approach for eye drug delivery as they can improve the corneal absorption [8]. In the past, some authors of our research group demonstrated the ability of SLN to increase the ocular bioavailability of topically-instilled antibiotics [9,10]. In addition, from the industrial viewpoint, the feasibility of sterilization by autoclaving, long-term stability, and easy large-scale production, allows to consider these systems as an important advance in the pharmacological treatment of eye disorders. 

However, to our knowledge, only a few descriptions reported the development of SLN for the delivery of drugs to the back of the eye [11] and this field is definitely worthy of further exploration.

On the other hand, nano- and microemulsion (µE) are widely studied in ophthalmology as delivery vehicles for drugs to the anterior and posterior sections of the eye [12,13,14]. The rationale of the use of µE as ocular drug delivery system lies on several advantageous physico-chemical properties: indeed, they are thermodynamic stable, show phase transition to liquid-crystal state, have very low surface tension and small droplet size, and present supersolvent characteristics due to the presence of both an aqueous and an oil phase, surfactant and co-surfactant mixtures. All these properties might positively influence some biopharmaceutical behaviours, improving drug retention and action duration, ocular absorption and drug permeation [15].

Endophthalmitis, although rare, is one of the most devastating complications of glaucoma surgery and cataract removing, involving internal eye structures that in an ageing population consists of a large fraction of ophthalmic operations [16]. Multiple measures for preventing endophthalmitis following cataract surgery have been studied until now. CEF is one of the widely used antibiotics to prevent post-operative bacterial endophthalmitis. High-certainty evidence shows that intracameral injection of 1 mg CEF lowers the chance of endophthalmitis after surgery, and there is evidence that using antibiotic eye drops in addition to CEF injection lowers the chance of endophthalmitis compared with using injections or eye drops alone [17]. Although intracameral administration is the most frequently used, recent reports [18] showed debilitating ocular toxicity caused by an accidental dosing error of CEF in patients undergoing cataract surgery; moreover, a transient macular oedema and diminished visual acuity was reported in 46% of the treated eyes following a single-dose intracameral injection of 9 mg of CEF [19]. For these reason, some authors [20] evaluated single-dose pharmacokinetic disposition of CEF after intravitreal, intracameral and topical administration in various eye tissues: the best results were obtained after intravitreal dosing of CEF in terms of higher ocular tissue distribution of the drug.

Moreover, over the years, intravitreal administration of antibiotics has become the mainstay of endophthalmitis management, which has evolved from the usage of systemic antibiotics in the past to the current use of intravitreal antibiotics, paving the way for nanotechnology in drug delivery in the future. Successful management of endophthalmitis could be enhanced by better understanding of pharmacokinetics of intravitreal antibiotics.

The aim of the present research is to develop a slow release CEF delivery system suitable to improve the ocular bioavailability (up to one week) of CEF avoiding patient discomfort due to the repeated postoperative administration of antimicrobial eye-drops after cataract surgery. Two different nanocomposite systems, based either on SLN or µE, thickened with a thermosensitive hydrogel (Pluronic®F127), are herein proposed and their ability to slow the release of the entrapped drug is evaluated. The in situ gelling formulations were prepared by adding Pluronic®F127 either to the SLN dispersion or to the µE, both loaded with dCEF prepared by esterifying CEF with 1-dodecanol in order to obtain a lipophilic CEF prodrug to be entrapped in the lipid matrix of SLN or dissolved in the disperse phase of oil in water (O/W) µE.

The need to predict *in vitro* drug and nanocomposite thermoreversible hydrogels clearance and distribution in the eye, stimulated the authors to develop a three-compartment eye cell.

## 2. Materials 

Cefuroxime sodium salt (CEF), 3-[bis(dimethylamino) methyl]-yl-3H-benzotriazol-1-oxide hexafluorophosphate (HBTU), dimethylformamide, Pluronic®F127 (PF127), Pluronic F68®(PF68) benzyl alcohol, n-dodecan-1-ol Sepharose®CL 4B, ethylacetate (EA), trilaurin, sulforhodamine B (SRB), dimethyl sulfoxide (DMSO), Triton®X-100 (octoxinol 9), pH 7.4 phosphate-buffered saline solution (PBS): 137 mM NaCl, 3 mM KCl, 80 mM Na_2_HPO_4_·2(H_2_O), 2 mM KH_2_PO_4_, trichloroacetic acid, dichlorometane, methanol, acetic acid, Sudan III (C.I. 26100), foetal calf serum and antibiotics for cell cultures were from Sigma Aldrich (St. Louis, MO, USA). High performance liquid chromatography (HPLC) grade acetonitrile, isopropyl myristate (IPM) taurocholic acid sodium salt, were from Fluka (Buchs, Switzerland). Epikuron®200 (soybean lecithin, containing 95% phosphatidylcholine) was from Lucas Meyer (Hamburg, Germany). Tween®80 (polisorbate 80), Tween®20 (polisorbate 20), and Cremophor®RH60 (PEG-60 hydrogenate castor oil) were from Acef (Piacenza, Italy). Brilliant Blue (C.I. 42660) was from Variati (Milano, Italy). Agar was from Alfa Aesar (Ward Hill, MO, USA). Hyasol®-BT (hyaluronic acid 1% *w*/*v*) was from Pentapharm (Basel, Switzerland). The retinal pigment epithelial ARPE-19 cell line (ATCC-CRL-2302) and the DMEM:F12 medium were purchased from LGC Standards (Middlesex, UK).

## 3. Experimental Methods

### 3.1. Synthesis and Characterization of Dodecyl-Cefuroxime (dCEF)

The derivative dCEF was synthesized by esterification with n-dodecan-1-ol of CEF sodium salt. ^1^H and ^13^C-NMR spectra were recorded on a Jeol 600 (Jeol Peabody, MA, USA) at 600 and 150 MHz, respectively. Chemical shifts (δ) are given in parts per million (ppm). The following abbreviations are used to designate the multiplicities: s = singlet, d = doublet, dd = doublet of doublets, m = multiplet. Low resolution mass spectra were recorded on a Micromass Quattro microTM API (Waters Corporation, Milford, MA, USA) with electrospray ionization. Melting points (mp) were determined with a capillary apparatus (Büchi 540). Flash column chromatography was performed on silica gel (Merck Kieselgel 60, 230–400 mesh ASTM). The progress of the reactions was followed by thin layer chromatography (TLC) on 5 × 20 cm plates with a layer thickness of 0.2 mm.

### 3.2. High-Performance Liquid Chromatography (HPLC) 

A reversed-phase column Allsphere ODS-2 5μ 150 mm × 4.6 mm (Alltech Italia, Milano, Italy), was used; UV–VIS detector was set at 279 nm and a constant flow rate of 1 mL/min was employed [21]. dCEF: CH_3_CN/H_2_O (90/10 *v*/*v*) was used as mobile phase; the retention time was 4.2 min. CEF: H_2_O/CH_3_CN/CH_3_COOH (74.9:25:0.1 *v*/*v*) was used as mobile phase; the retention time was 6.3 min.

### 3.3. Aqueous Stability of CEF and dCEF and In Vitro dCEF Hydrolysis

The stability of CEF and dCEF in aqueous solution was assessed. Solutions of CEF and dCEF at concentration of 0.1 mg/mL in 15% *v/v* Tween®80 were prepared and then stored at 4 °C for one month or incubated at 35 ± 0.5 °C for seven days. At scheduled intervals, the concentration was determined by HPLC and then expressed as the ratio, in percentage, of the sampling time concentration vs. the initial one (Ct/C_0_ × 100).

To evaluate the rate of hydrolysis of dCEF in presence of porcine liver esterase, in vitro tests were set up. Aliquots of dCEF dissolved in acetone in the presence of different surfactant solutions were prepared as follows:

-dCEF (0.03 and 0.3 mg/mL) in a mixture of Triton 0.5 mg/mL and Tween®80 0.5 g/L.

-dCEF (0.03 and 0.3 mg/mL) in Tween®80 0.5 g/L.

All samples were dried under nitrogen flow and added with 99 µL of 100 mM, pH 8 phosphate buffer; the resulting suspensions were then thoroughly vortexed in order to form micelles and afterwards 1 µL porcine liver esterase (equivalent to 5 U enzyme) was added. 

Thereafter samples were incubated for 1 h at 35 °C and the concentration of free CEF was determined by HPLC and expressed as the percent ratio of CEF released from the hydrolysis of dCEF (CEFf) *versus* the initial concentration of dCEF (dCEFi) in the different incubation media (CEFf/dCEFi)%.

In a second step, the experiment was repeated on 0.03 mg/mL dCEF with 10 µL liver esterase (50 U) for either 1 or 2 h of incubation time.

### 3.4. Preparation of IPM-Based µE (µE1)

The basic method of preparing an O/W µE consists in dissolving a selected surfactant in the oil to be emulsified in an amount effective to yield a fine emulsion. The lipid mixture is then added to the water phase and shaken or stirred. Finally, a second surfactant is added which is somewhat more soluble in water than the first surfactant to produce a substantially clear system. 

In the present research, in agreement with our previous investigations [22], IPM was used as lipid phase, Epikuron®200 as surfactant, taurocholic acid sodium salt and benzyl alcohol as cosurfactants. µE1 (O/W) was obtained as follows: dCEF was solubilized in IPM and Epikuron®200, and the mixture was progressively enriched with distilled water, added dropwise under stirring. Taurocholic acid sodium salt was then added vortexing for some minutes; afterwards benzyl alcohol was introduced under stirring up to transparency. 

### 3.5. Preparation and Characterization of Solid Lipid Nanoparticles (SLN)

Blank SLN and dCEF-loaded SLN (dCEF-SLN), were prepared with the method called *cold microemulsion dilution* [23,24]. A solution of trilaurin in EA was used as lipid phase, while Epikuron®200, taurocholic acid sodium salt and Cremophor®RH60 were employed as surfactant/cosurfactant. EA and water were mutually pre-saturated (solubility of EA in water: 8,7 g/100 mL) before use. Briefly, the O/W µE (µE2) was obtained by mixing appropriate amounts of oil, surfactant, cosolvent/cosurfactant and adding EA-saturated water drop by drop to the lipid phase by vortexing at room temperature up to transparency. µE2 was then diluted with an aqueous solution of PF68 (2% *w*/*v*) to precipitate SLN.

SLN particle sizes, polydispersion indices (PDI) and *ζ* potential were determined using dynamic light scattering (DLS) technique (LLS, Brookhaven, NY, USA). Measurements were obtained at fixed angle of 90° at 25 °C. All data were determined in triplicate.

The homogeneity of the suspension was checked with optical microscopy (DM2500, Leica Microsystems, Wetzlar, Germany).

Particle shape was determined through scanning electronic microscopy (SEM-Stereoscan 410, Leica Microsystems, Wetzlar, Germany): a few drops of SLN were placed on aluminium stub, left to dry under vacuum for one night and then metallized with graphite to increase their conductivity. 

The formation of SLN was further assessed by DSC analysis. A Q200 (TA instruments, New Castle, DE, USA) differential scanning calorimeter was used to determine melting temperatures (Tf) of bulk lipid (trilaurin), bulk drug (dCEF), dCEF-SLN dispersion. Measurements analyses were performed in the 20–150 °C temperature range, with a scanning rate of 10 °C/min and under a nitrogen flow of 50 mL/min. Sealed aluminum pans containing ~5 mg of freeze-dried samples were used; an empty aluminum pan was used as a reference.

Entrapment efficiency (EE%) was determined by a size exclusion method. 1 mL SLN underwent gel filtration using a matrix of cross-linked agarose (Sepharose®CL4B) as stationary phase and PBS, pH 7.4 as mobile phase. The opalescent fractions containing the purified SLN were concentrated under nitrogen up to 1 mL final volume. The amount of dCEF in the resultant suspension was determined solubilizing 0.05 mL SLN into 0.95 mL CH_3_CN and analysing it by HPLC. In this case, EE% was calculated as the ratio between the drug recovery after and before gel filtration.

### 3.6. Preparation of Nanocomposite Hydrogels

µE1-based thermosensitive nanocomposite hydrogel (M-TNH) were prepared as follows: a weighted amount of PF127 (14% *w*/*v*) was added to the µE1 to obtain M-TNH.

SLN-based thermosensitive nanocomposite hydrogel (S-TNH) were prepared as follows: SLN were prepared from µE2 with the above reported method; after precipitation, a weighted amount of PF127 (20% *w*/*v*) was added to the SLN aqueous dispersion to obtain S-NTH.

In both case the resulting pH was 7.2.

### 3.7. Stability Studies 

The physico-chemical stability of µE1 and M-TNH was monitored over a period of six-month storage at 4 °C by visual observation and pH determination. Additionally, the physico-chemical stability of blank SLN, dCEF-SLN and dCEF-S-TNH was assessed by monitoring weekly the average size and *ζ* potential values, over a period of one-month storage at 4 °C. 

At the same time intervals, also the chemical stability of dCEF loaded both in SLN and in S-TNH was assessed at 4 °C: after proper sample dilution, dCEF content was determined by the previously described HPLC method.

### 3.8. Syringeability and Gelation Tests

S-TNH and M-TNH were aspired and then injected through a syringe with a 30-gauge needle and qualitatively assayed for syringeability (easy, moderate, challenging, fail).

Gelation of S-TNH and M-TNH at physiological intraocular temperature (35 °C) was initially determined by the test tube inverting method [25]. An aliquot (1.0 mL) of each sample was prepared at 10 °C in a glass tube and then placed into a thermostatic incubator at 35 °C. Gelation was considered to occur when a gel-like material was obtained that did not exhibit gravitational flow during a period of 2 min when the tube was reversed.

### 3.9. Viscosity and Rheological Studies 

The viscosity behaviour and rheological properties of the hydrogel (PF127 20% *w*/*v* solution) and of S-TNH and M-TNH were examined by a rheometer Discovery HR1 (TA Instruments, New Castle, DE, USA) equipped with cone/plate geometry (diameter 40 mm, cone angle 1°, truncation 28 μm). Temperature control was obtained by a Peltier plate. 

Measurements in oscillatory mode were performed to obtain the gel point with a temperature ramp from 10 to 45 °C with a heating rate of 10 °C/min. Samples were equilibrate at 10 °C for 900 s. Stress was set at 0.2 Pa and frequency at 1.0 Hz. Viscosity measurements were performed in flow ramp mode. Duration of the measure was 120 s, ranging from 0 to 100 s^−1^. Measurements were done at 10, 25 and 35 °C, equilibrating the samples for 900 s at the set temperatures.

### 3.10. Ocular Flow Cell

#### 3.10.1. Development of Ocular Flow Cell 

Recently, Awwad et al. [26] developed a two-compartment in vitro eye flow model, called PK-Eye, for the estimation of ocular drug clearance by the anterior aqueous outflow. The model was designed to mimic the mass transfer characteristics due to anterior aqueous outflow and was proposed as a tool in preclinical studies to accelerate the development of slow release drug delivery systems aimed to treat chronic eye diseases.

**Figure 1:**
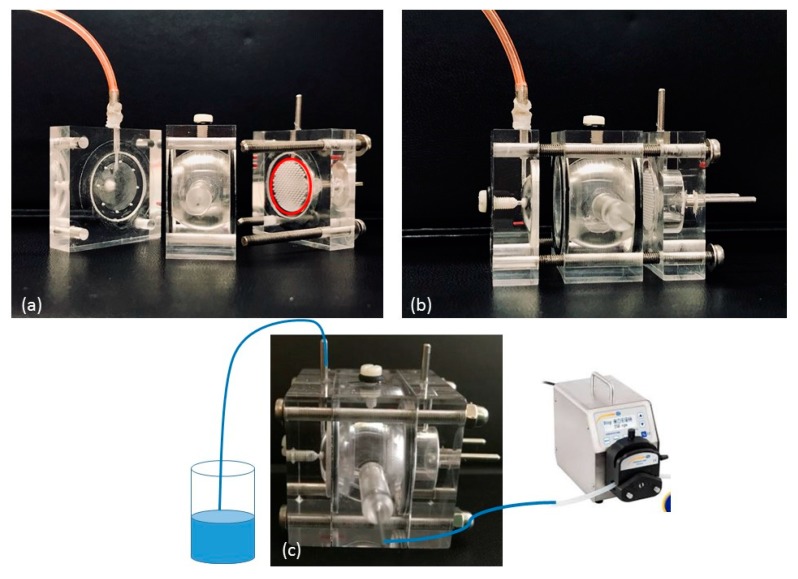
Ocular flow cell: (**a**) the separated three-compartments and the semipermeable disk, possible support for retinal cells (red circled); (**b**) the assembled flow cell with screw-secured compartments; (**c**) the cell connected with the dispensing peristaltic pump.

Starting from, and partially modifying this model, we designed and developed a Plexiglas three-compartment eye flow cell (Figure 1). The model comprises three pieces secured together with four screws. The anterior and posterior cavities of the flow cell, mimicking the anterior and the posterior sections of the eye respectively, are separated by a porous separator disk on which a membrane can be placed. The two cavities were designed to be a similar in size as in the human eye (0.25 mL and 7.75 mL, respectively). In addition, a semipermeable disk is placed vertically in the posterior section towards the posterior wall: the idea is to divide the posterior section of the cell in a central unit, acting as the vitreous cavity (6.65 mL) and in a posterior unit (1.1 mL); moreover, it can be used as a support for retinal cell cultures to mimic the blood retinal barrier. In this way, three compartments can be clearly identified in our model. An injection port is placed at the top of the vitreous cavity; the aqueous inlet port is placed in the vitreous cavity near the membrane barrier, whilst an outlet port is placed in the anterior cavity. Both ports have 1.5 mm ID.

#### 3.10.2. Simulated Vitreous Preparation

Simulated vitreous was prepared according to literature [27]. Briefly, agar (0.4 g) was dissolved in 100 mL hot water and then carefully vortexed under IKA T-25 Ultraturrax (IKA®-Werke GmbH, Staufen D). Separately 50 mL of Hyasol-BT (hyaluronic acid (HA) (0.5 g) were dispersed in 100 mL water. Equal volumes of both solutions were then mixed for 5 min under Ultraturrax to obtain a homogenous medium to which a few drops of 0.02% *w*/*v* sodium azide were added. The mixture was cooled to room temperature (20 °C) until a gel-like consistency was reached.

Viscosity measurements were performed at 35 °C employing a coaxial cylinder type rotational viscometer (Programmable DV-III + Rheometer, Brookfield, Middleboro, MA, USA) at 0–34 s^−1^ shear rate; spindle n. 31. Viscosity measurements were repeated also after seven- and 14-day storage at 35 °C. Each measurement was repeated thrice.

#### 3.10.3. Dye Clearance Studies 

The whole cell was filled by simulated vitreous and the inlet port of the cell was connected with tubing (1.5 mm ID) to a dispensing peristaltic pump (Minipuls^®^3, Gilson, Middleton, WI, USA) that provided aqueous NaCl 0.9% *w*/*v* at 2.0 µL/min inflow. 

A total of 100 µL of each sample under study was injected through the injection port at a temperature not higher than 15 °C and the cell was then placed in a thermostatic incubator (MIR-154-PE, Panasonic, Milano, Italy) at 35 ± 0.5 °C.

The following systems were injected in separate experiments:

0.1% *w/v*-Brilliant Blue aqueous solution.

0.1% *w/v*-Brilliant Blue in 20% *w*/*v* PF127 aqueous solution.

0.1% *w/v*-Sudan III in SLN.

0.1% *w/v*-Sudan III in S-TNH.

Clearance experiments were followed up to one week for both the dyes. Images were obtained using a digital camera at various time points to visualize the dye clearance from the model.

### 3.11. In Vitro Release

Two distinct in vitro release methods were employed to evaluate the release of dCEF from the selected nanocomposite formulations.

In the first method, the formulations were introduced in a tube at 35 °C with the receiving phase, from which samples were withdrawn at prefixed time intervals.

In the second method, drug release was monitored using the ocular flow cell perfused with buffer solution by a precision pump controlling the in/out flow rates. 

(a) Tube Release Test

Release studies were performed at 35 °C using a tube test, according to which a fluid receiving phase (8 mL, 15% *v/v* Tween®80 in PBS) is layered onto the surface of the donor phase (1 mL). At scheduled times, 0.2 mL of the receiving phase was collected, centrifuged and the supernatant analyzed by HPLC.

The systems under study were the following: dCEF-SLN (0.33 mg/mL equivalent to 0.25 mg/mL); dCEF-S-TNH (dCEF 0.33 mg/mL equivalent to CEF 0.25 mg/mL); dCEF-M-TNH (dCEF 1 mg/mL) and CEF (1 mg/mL) in 20% *w/v* PF127 hydrogel as references. The actual dCEF concentration was calculated according to EE%.

(b) Release Studies by Ocular Flow Cell

In a similar way to dye clearance studies, the ocular cell was filled with the simulated vitreous, then 100 µL of each sample were introduced through the injection port and, maintaining the temperature at 35 °C, at scheduled time intervals samples were collected from the outflow of the anterior cavity and stored at 4 °C until analysis by HPLC. 

The systems under study were the following:

dCEF-SLN (dCEF 1 mg/mL equivalent to CEF 0.75 mg/mL); dCEF-S-TNH (dCEF 1 mg/mL equivalent to CEF 0.75 mg/mL); CEF (1 mg/mL) aqueous solution and CEF (1 mg/mL) in 20% *w/v* PF127 hydrogel as references.

A higher dCEF concentration in SLN and in S-TNH than in tube release test (1 mg/mL versus 0.33 mg/mL) was obtained by nanoparticles freeze-drying and resuspension in water.

Operative conditions were the same as described in Section 3.10.3, except the perfusion medium that was 15% *v/v* Tween®80 aqueous solution.

### 3.12. Cell Proliferation Assay

The biocompatibility of blank SLN, blank µE1 and of the gel matrix (20% *w/v* PF127) were evaluated on retinal pigment epithelial ARPE-19 cells by using a Sulforhodamine B colorimetric proliferation assay (SRB assay). 

ARPE-19 were routinely grown in DMEM:F12 medium, with the addition of 10% (*v*/*v*) foetal bovine serum, 1% (*v*/*v*) penicillin–streptomycin, and were maintained in standard conditions (37 °C, 5% CO_2_ and 95% humidity). 

For cell proliferation assay, blank SLN dispersion, blank µE1 and 20% *w*/*v* PF127 hydrogel were tested. Ten thousand cells were seeded into 96-well plates. After 24 h, cells were incubated for 72 h, in triplicate wells, with the two samples properly diluted (1:100, 1:200; 1:500, 1:700) in culture medium. The SRB assay was carried out as previously described [28,29,30]. The experiment was replicated twice for both incubation times.

### 3.13. Microbiological Studies

(a) Bacteria Isolation 

Corneal surface swabs were obtained by three patients recovered at the Molinette Hospital (Torino, Italy). Patients were enrolled according to the following inclusion criteria: corneal pathology that requires a surgical treatment, and no local or systemic drug administration in the previous three weeks. Samples were collected in a surgery room using sterile swabs with specific medium and stored at 4 °C until analysis. 

Samples were examined with a four-step procedure and they are as follows: pre-enrichment, enrichment, selective and differential culture media, and identification. Briefly, eye swabs passed on the corneal surface were subjected to pre-enrichment, and each sample was soaked in 9 mL buffered peptone water (BPW) medium for 30 s, and then stored at 20 °C. The BPW was incubated at 37 ± 1 °C for 18 ± 2 h. The enrichment step used 100 ± 10 mL of BPW dispersed in 10 ± 0.5 mL of Müller Kauffmann tetrathionate broth with novobiocin, this was incubated at 37 ± 2 °C for 24 ± 3 h. One mL of colony material from the opaque zone of the colonies was streaked on XLD + N and BGA using a sterile disposable loop and incubated at 37 ± 2 °C for 24 ± 2 h. The resulting colonies were suspected to be *Staphylococcus epidermidis*: these were inoculated into triple sugar iron and incubated at 37 °C for 24 h. Identification was performed using a commercial micromethod (API 20E; BioMerieux, Roma, Italy).

(b) In Vitro Antimicrobial Susceptibility Test

The microbiological evaluations were performed using Kirby Bauer and minimal inhibitory concentration (MIC) assays.

• Kirby–Bauer

Each strain was analysed to evaluate the antimicrobial susceptibility following the Kirby–Bauer method described in Clinical and Laboratory Standards Institute guidelines. Using a sterile inoculating loop or needle, material was collected from four or five isolated colonies of organisms; the colonies were then suspended in 2 mL sterile saline solution and the tube was vortexed to create a smooth suspension (adjusting the turbidity to 0.5 McFarland standards). A sterile swab was dipped into the inoculums solution and rotated against the side of the tube using firm pressure to remove the excess fluid. Müller-Hinton agar was inoculated by streaking the swab three times over the entire dried surface of the agar. Appropriate antimicrobial-impregnated disks were placed on the surface of the agar; forceps were used to dispense the antimicrobial disks one at a time. Then, all plates were incubated at 37 °C for 24, 48, 72 and 96 h. The Kirby–Bauer test protocol provides for the measurement of inhibition zone of microbial growth around the disk. Using a caliper, each zone was measured with the unaided eye while viewing the back of a Petri dish. The CEF pharmacological activity against bacteria strains was tested. On the recording sheet, depending on the inhibition zone size, the strain was classified as susceptible (S; >18 mm), or resistant (R; <18 mm), compared with the reference values.The same procedure was repeated with the following samples: blank SLN, blank S-TNH, dCEF; dCEF-SLN, dCEF-S-TNH.

• Minimal inhibitory concentration (MIC)

Minimal inhibitory concentration (MIC) was determined in broth using the microdilution method according to Clinical and Laboratory Standards Institute (CLSI) guidelines. Briefly, *Staphylococcus epidermidis* strains were seeded on Trypticase Soy Agar (TSA) (Oxoid, Milano, Italy). Colonies following overnight growth were directly suspended in Müller–Hinton broth (MHB) (Oxoid, Milano, Italy) to obtain a turbidity comparable to the McFarland turbidity standard of 0.5. Cultures were diluted 1:100 with broth to reach a final concentration of 106 colony forming units (CFU)/mL. CEF solution (standard HPLC Sigma-Aldrich St. Louis, MO, USA, final concentration of 64 μg/mL) was added either to MHB. Serial dilutions from this solution were prepared in broth to reach concentrations ranging from 32 μg/mL to 0.008 μg/mL, and in presence of approximately 5 × 105 CFU/mL. Plates were incubated at 37 °C 37 °C for 24, 48, 72 and 96 h and read at 600 nm, using an Ultraspec 2000 Spectrophotometer (Pharmacia Biotech, Milano, Italy). 

The same procedure was repeated with the following samples: blank SLN, blank S-TNH, dCEF; dCEF-SLN, dCEF-S-TNH.

### 3.14. Statistical Analysis

DLS data (diameters and *ζ* potential values) and percentage of CEF hydrolysis were expressed as means ± standard error. In the stability and release studies, comparisons among formulations were performed by a one-way analysis of variance (ANOVA), using Graph Pad Prism (v 6.01) software (San Diego, CA, USA). A value of *p* < 0.05 was considered statistically significant.

## 4. Results

### 4.1. Synthesis and Characterization of dCEF

**Figure 2:**
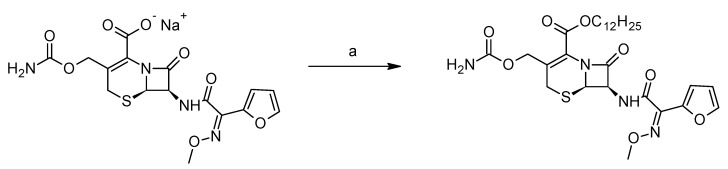
dCEF synthesis scheme: (**a**) n-dodecan-1-ol, HBTU, DMF, 25 °C, 18 h.

The derivative dCEF (IUPAC name: dodecyl(6R,7R)-3-[(carbamoylossi)methyl]-7-[(2E)-2-(furan-2-yl)-2-(methoxyimino)acetamido]-8-oxo-5-tia-1-azabicyclo-[4.2.0]ott-2-ene-2-carboxylate; molecular weight 592.70 g/mol) was synthesised by esterification with n-dodecan-1-ol of the CEF sodium salt in the presence of 3-[bis(dimethylamino) methyl] -yl-3*H*-benzotriazol-1-oxide hexafluorophosphate (HBTU) as a coupling agent (Figure 2). 

Briefly, in a 50 mL flask, CEF sodium (0.11 mmol) was placed in 5 mL of dimethylformamide. The solution was cooled in an ice bath, then n-dodecan-1-ol (1.1 eq) and HBTU (1.1 eq) were added. The mixture was stirred at room temperature for 18 h, then the solvent was evaporated under nitrogen flow and the crude was purified by flash chromatography on silica gel, eluting with dichloromethane/methanol 98/2 (pale yellow solid, yield 46%).

The lists of 1H and 13C NMR peaks are reported below, whilst the spectra have been included as Supplementary Materials: Figures S1 and S2.

M.p.: 129.1–130.5 °C. MS ESI-: 591 [M-1]-. 1H-NMR (CDCl3) 7.52 (m. 1H arom), 7.33 (m, 1H), 6.92 (d, *J* = 3.4 Hz, 1H), 6.49 (dd, *J* = 3.4, 1.7 Hz, 1H), 5.82 (dd, *J* = 4.1, 8.6 Hz, 1H), 5.39 (d, *J* = 4.1 Hz, 1H), 5.08 (d, *J* = 1.4 Hz, 1H), 4.67 (m, 4H), 4.2 (m, 2H), 4.11 (s, 3H), 1.69 (m, 2H), 1.27 (br. S, 18H), 0.89 (m, 3H). 13C-NMR (CDCl3) 167.1, 163.7, 160.3, 160.1, 156.1, 145.4, 144.7, 142.9, 122.0, 119.5, 114.6, 111.8, 66.7, 65.9, 63.5, 60.1, 53.3, 50.0, 31.8, 29.6, 29.5, 29.4, 29.3, 29.1, 28.4, 25.7, 22.6, 14.1 

### 4.2. Stability of CEF and dCEF in Aqueous Solution 

The beta-lactam ring structure is essential to microbiologic activity; however, it is unstable when exposed to water: hydrolysis of the ring occurs resulting in the loss of activity. Additionally, knowledge of drug stability is important to ensure that the correct dose is administered [31].

The stability of cephalosporins in aqueous solutions was formerly investigated and according to the literature, the optimum pH range of stability was determined to be 4.5–7.3 [32]. In aqueous solution CEF was stable for one day (more than 90% potent) at 25 °C and for at least 30 days at 5 °C [33].

According to these literature data, in the present work we investigated and compared the stability of CEF and dCEF in aqueous solutions at 4 ± 1 °C for one month to mimic the storage conditions. Moreover, considering that the goal we are aiming with the novel formulations here proposed is to prolong the antibiotic coverage after intravitreal administration of a single dose up to seven days, a stability study was also planned at the physiological intraocular temperature of 35 ± 0.5 °C over a period of one week.

**Figure 3:**
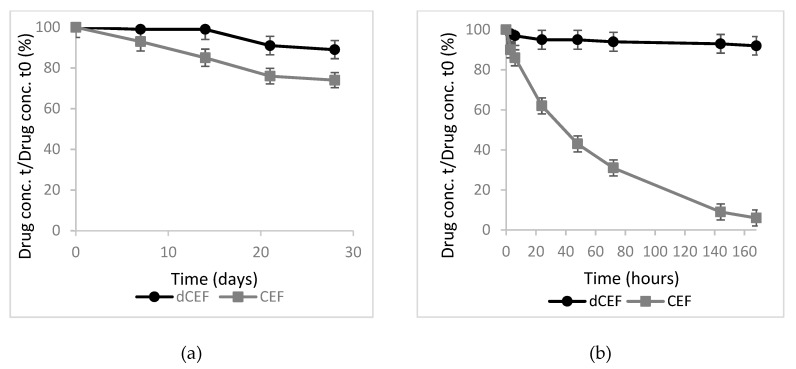
Stability of CEF (grey) and dCEF (black) in 15% *v/v* Tween®80 (each bar represents the mean ± SD; *n* = 5); (**a**) at 4 °C, for one month: from the 2nd to the 4th weeks dCEF resulted significantly more stable then CEF stored in the same conditions (*p* < 0.05); (**b**) at 35 °C, for one week: from 24 h dCEF resulted significantly more stable then CEF (*p* < 0.01 to < 0.001).

A micelle-forming surfactant was necessary to solubilize lipophilic dCEF in water, and, to compare the degradation profiles of CEF with dCEF in the same medium, both products were dissolved in 15% *v/v* Tween®80 aqueous solution. When stored at 4 °C, a 10% reduction of CEF initial concentration was noted after three days, while dCEF was stable for two weeks (Figure 3a). When both were incubated 35 ± 0.5 °C a significant difference (*p* < 0.001) was noted: after 24 h CEF was subjected to 40% reduction of its initial concentration, meanwhile dCEF was stable for one week (Figure 3b). Thus dCEF, being more stable than CEF, probably as a result of the lipophilic chain that prevents hydrolytic phenomena, can be reasonably proposed both as lipophilic and more stable CEF derivative.

### 4.3. In Vitro dCEF Hydrolysis

The aim of this assay was to verify whether CEF can be released from dCEF by cleavage of the ester bond by a non-specific esterase: as it is well-known that the vitreous humor has esterases and hydroxylases [34], we hypothesized that a sufficient amount of free CEF might be released from its lipophilic prodrug dCEF, necessary precondition to ensure its antibiotic activity after intravitreal administration.

When hydrolysis was performed with 5 U porcine esterase CEF_f_/dCEF_i_ % was low, especially in the presence of Triton®X-100: in fact, in the absence of Triton®X-100, hydrolysis increased of an order of magnitude at both the enzyme/dCEF ratios (Table 1).

For this reason, the experiment was repeated with an excess of enzyme (50 U) prolonging the incubation time up to 2 h (data not shown): after 1 h, CEF_f_/dCEF_i_ % was 8.7 and increased up to 11.7 after 2 h incubation time. Further *in vivo* experiments will be needed in future studies to confirm the real hydrolysis of dCEF.

### 4.4. Formulation of µE

The obtained µE1 had the following composition: IPM (85 mg), Epikuron®200 (70 mg), Tween®20 (88 mg), taurocholic acid sodium salt (40 mg), benzyl alcohol (40 mg), water (700 mg).

It resulted isotropic under polar light observation, and was transparent to visual observation up to more than six-month storage at room temperature.

### 4.5. Formulation of SLN 

Blank and dCEF-loaded SLN (dCEF-SLN) were obtained using the *cold microemulsion dilution* technique: the lipid matrix, dissolved in a partially water-soluble solvent used as µE2 oil phase, precipitates upon µE dilution with a polymeric stabilizer aqueous solution, which allows the formation of spherical nanoparticles. This technique is considered an evolution of a method previously developed by our research group which employed O/W emulsions [24,35]. It combines the advantages of the emulsion solvent diffusion technique with the high stability and the super-solvent properties of microemulsive systems. It does not require extreme operative conditions, such as high/low temperatures, pH modification, pressure variations, the use of toxic solvents, and/or the application of ultrasonication or homogenization, all conditions that might undermine drug stability. The obtained µE2 had the following composition: Trilaurin (60 mg), Epikuron®200 (170 mg), ethyl acetate pre-saturated with water (180 mg), Cremophor®RH60 (53 mg), taurocholic acid sodium salt (10 mg), water pre-saturated with ethyl acetate (700 mg).

The precipitation of trilaurin SLN from the O/W µE (µE2) was obtained after its dilution with a 2% *w*/*v* PF68 aqueous solution: SLN are, therefore, mainly composed by trilaurin and lecithin (Epikuron®200), both recognized as biocompatible substances, so we can predict they can be suitable to be proposed as safe nanosystems for drug delivery.

In dCEF-SLN preparation, the previously described µE2, containing 2 mg dCEF (corresponding to 1.5 mg CEF) dissolved in the oil phase, was diluted by a 2% *w*/*w* solution of PF68 in the same way, the final composition is reported in Table 2.

### 4.6. Characterization of SLN 

SLN suspensions were prepared and checked in triplicate. 

Mean diameters (± standard error, S.E.), PDI and *ζ* potential of blank SLN and dCEF-SLN were determined by DLS technique and are reported in Table 3.

dCEF-SLN displayed a mean diameter around 330 nm, which was higher than that of blank SLN (around 280 nm). Thus, it can be partially ascribable to the purification process and/or to the entrapment of the drug into SLN, which might modify in some way the lipid matrix organization. 

*ζ* potential values are stabilized around −20 mV, and they are reasonably due to the presence of anionic surfactants and co-surfactants on the surface of the particles; moreover, they could indicate a certain stability of the SLN dispersion. The high EE% value, obtained by gel filtration, confirmed the possibility to entrap a hydrophilic substance as CEF in SLN modifying its structure by esterification. 

**Figure 4:**
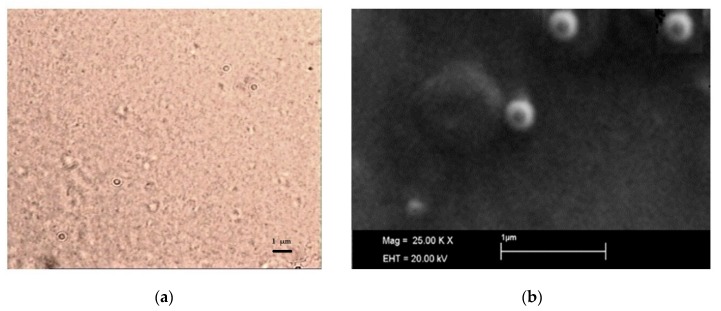
Images of dCEF-SLN: (**a**) optical microscopy (630×); (**b**) SEM (25,000×).

SLN micrographs obtained by optical microscopy are reported in Figure 4: optical microscopy (Figure 4a) allowed excluding the presence of microparticles or drug crystals and, even if it was not possible to clearly visualize the single nanoparticle, a homogeneous nanosuspension was observed. 

SEM observation confirmed the particle size detected by DLS (Figure 4b) and assessed the spherical shape of SLN. 

**Figure 5:**
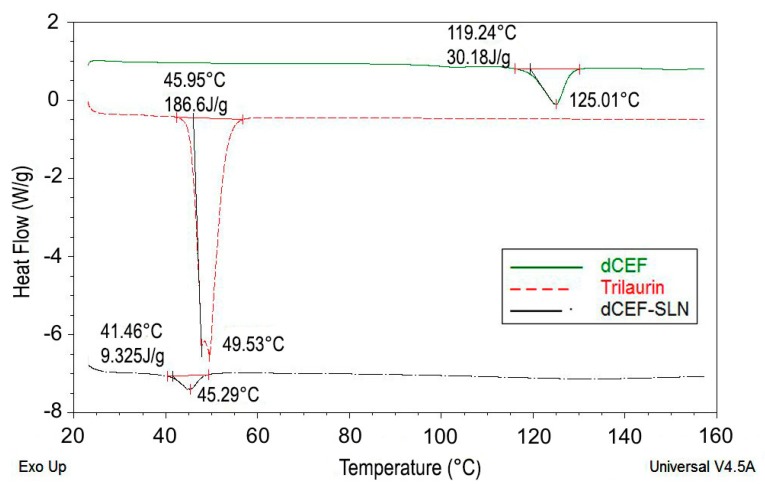
DSC thermograms of raw dCEF (upper solid curve), raw trilaurin (intermediate dotted curve) and dCEF-SLN (lower dash-dotted curve).

The formation of SLN was further assessed by DSC analysis. Particularly, DSC was used to analyse bulk lipid (trilaurin), bulk drug (dCEF) and dCEF-SLN dispersions. Both trilaurin and dCEF had different thermal behaviours when entrapped in SLN or when present as raw materials. As shown in Figure 5, DSC thermograms of SLN revealed a sharp melting peak of trilaurin, which was slightly shifted towards lower temperatures. According to Siekmann and Westesen [36], the melting point decrease of SLN colloidal systems can be due to the colloidal dimensions of the particles, in particular to their high surface-to-volume ratio, and not to recrystallization of the lipid matrices in a metastable polymorph. If the bulk matrix material is turned into SLN, the melting point is depressed and the presence of impurities, surfactants and stabilizers could also affect this phenomenon [37,38].

Similarly, when dCEF was SLN-entrapped, it was no more possible to evidence a peak corresponding to dCEF melting point, as probably, during the formation of SLN and the simultaneous entrapment of dCEF, trilaurin may partially inhibit the crystallization of dCEF. These results confirm dCEF entrapment in nanoparticles.

### 4.7. Stability Studies

Stability studies were conducted: (i) to define the physico-chemical stability of SLN, dCEF-SLN and dCEF-S-TNH and (ii) to evaluate the chemical stability of dCEF loaded in M-TNH, SLN and S-TNH. The study was performed for one month at 4 °C to reproduce long-term storage conditions. 

**Figure 6:**
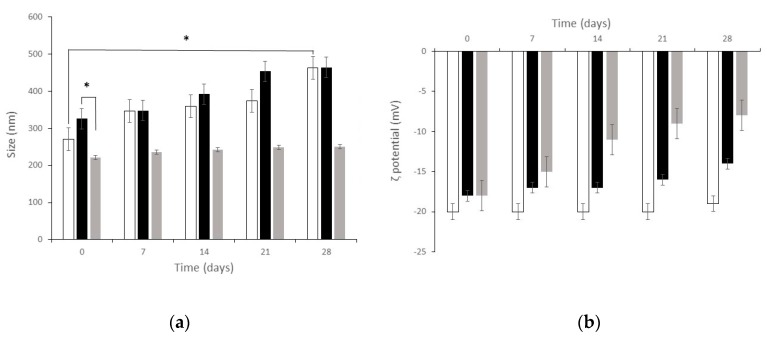
Physico-chemical stability at 4 °C of blank SLN (white) and of dCEF-SLN (grey) and dCEF-S-TNH (black) in term of: (**a**) mean size and (**b**) *ζ* potential. Each bar represents the mean ± SD (*n* = 5); * (*p* < 0.05).

Figure 6 shows particle mean diameter and *ζ* potential of both blank SLN and dCEF-loaded SLN (dCEF-SLN and dCEF-S-TNH) over four-week storage at 4 °C. As regards blank SLN, at time, mean diameter was 271 ± 9.2 nm with PDI of 0.285, and *ζ* potential was −20 ± 1.6 mV. After four-week storage, mean diameter was 463 ± 9.2 nm with PDI of 0.342, and *ζ* potential was −19 ± 2.8 mV with a significant increase in mean diameter, probably due to aggregation phenomena over time. Mean diameters of dCEF-S-TNH should be considered as SLN diameters when SLN are dispersed in a thermosensitive gel of PF127: they were significantly lower than those of dCEF-SLN, probably as the presence of PF127 in the disperse phase and onto SLN surface can prevent aggregation. 

Globally, these results demonstrate the suitable stability of particles with just a low decrease of the recorded values suggesting a certain degree of long-term stability.

**Figure 7:**
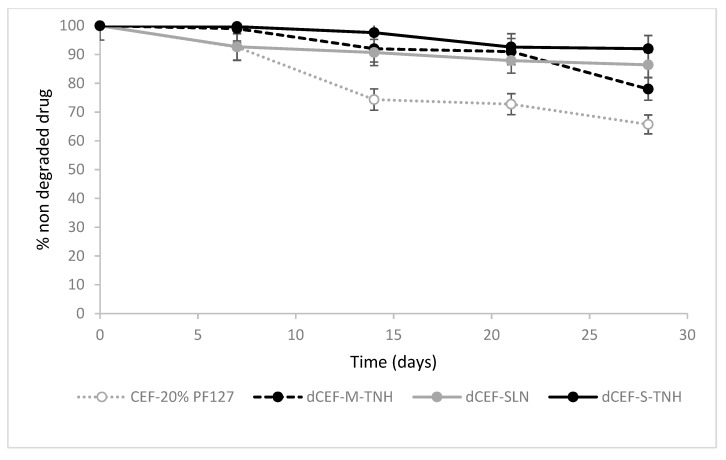
Long-term chemical stability upon storage at 4 °C of: CEF in 20% *w*/*v* PF127 hydrogel (dashed grey), dCEF-M-TNH (dashed black), dCEF-SLN (grey), dCEF-S-TNH (black). Each bar represents the mean ± SD (*n* = 5). Significant differences versus CEF reference started from the 15th days for the dCEF-preparations (*p* < 0.05), among the dCEF preparations no significative differences were noted during 28 days (*p* > 0.05).

The overtime-chemical stability of dCEF was then investigated at 4 °C in SLN and in both the nanocomposite systems, SLN-S-TNH and M-TNH, in comparison with the chemical stability of CEF dissolved in 20% *w*/*v* PF127 as reference (Figure 7).

In term of drug chemical stability, no significant differences were detected between dCEF-SLN aqueous dispersion, dCEF-S-TNH and dCEF-M-TNH (*p* > 0.05): in all media dCEF was fairly stable for 28 days at 4 °C. 

The stability of free lipophilic dCEF was not investigated in pure hydrogel because of its low solubility, thus only CEF stability in hydrogel was monitored as reference. Namely, the decrease after one-month storage at 4 °C resulted of 13.6% and 7.1% in SLN and in S-TNH, respectively, meanwhile, over the same period, CEF concentration decreased of 34.3% in PF127 hydrogel. Therefore, it can be considered that SLN, and even more their dispersion in hydrogel, improve the stability of CEF in its esterified form.

### 4.8. Syringeability and Gelation Tests 

Syringeability and injectability are product performance key parameters of any parenteral dosage form. The former refers to the ability of an injectable therapeutic to pass easily through a hypodermic needle on transfer from a vial prior to an injection, while the latter refers to the performance of the formulation during injection [39].

Additionally, gelation is a key factor in this type of system that should create a depot to prolong the entrapped drug release. The commonest diagnostic test of gelation is to turn a test-tube or vial containing the sample upside-down and then to note whether the sample flows under its own weight. 

**Figure 8:**
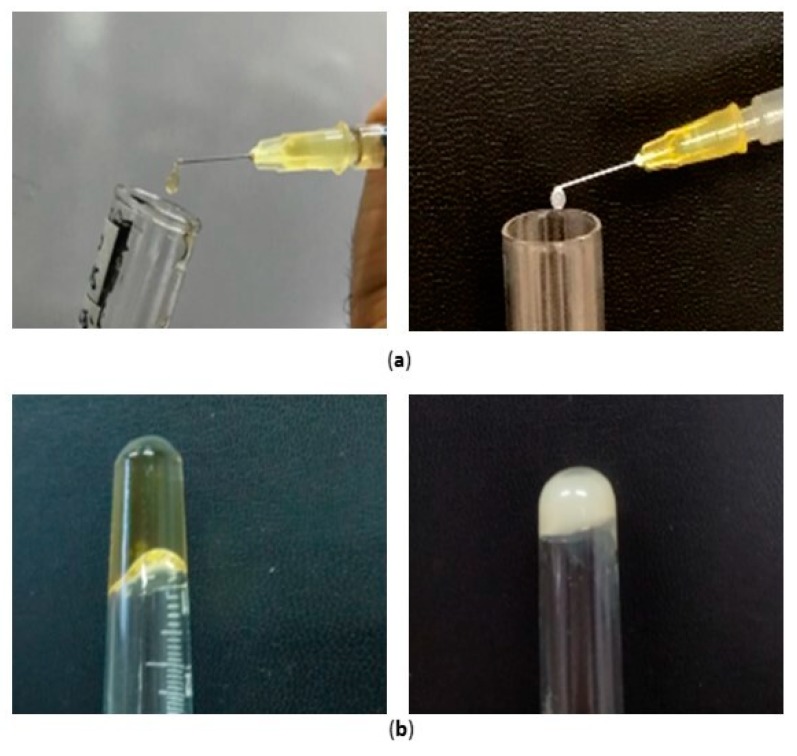
Photographs showing: (**a**) the syringeability of M-TNH (left) and S-TNH (right) at room temperature; (**b**) the gelation at 35 °C of M-TNH (left) and S-TNH (right).

The syringeability of both the nanocomposite systems, namely S-TNH and M-TNH, was manually assessed by the employment of a syringe (Figure 8a). As the needle size might influence the syringeability, in this study a needle consistent with intraocular injections (30 gauge) was used, revealing the suitability of both formulations to be injected intravitreally. Moreover, both the systems S-TNH and M-TNH satisfied the tube inversion test (Figure 8b), confirming the thickening of the nanocomposite systems at physiological temperature. 

Since it is well recognized that apparent viscosity deeply affects the ejection of a formulation from the syringe via a needle to the injection site, rheological measurements were subsequently performed to deeply investigate the behaviour indicated by tube inversion.

### 4.9. Viscosity and Rheological Studies

**Figure 9:**
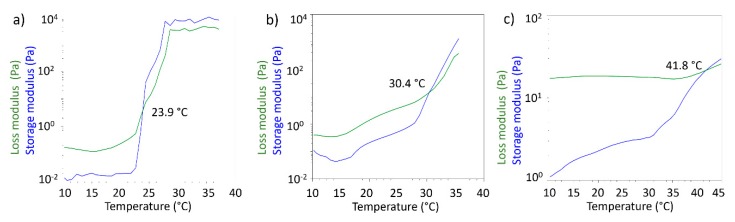
Storage modulus (G’) and loss modulus (G’’) as a function of temperature of: (**a**) 20% *w*/*v* PF127 aqueous solution; (**b**) S-TNH; (**c**) M-TNH.

Rheological measurements performed in oscillatory mode allowed to obtain storage modulus (G’) and loss modulus (G’’) as a function of temperature. G’ and G’’ are indicators of the viscoelastic behaviour of the tested formulations, and in particular it is known that when G’ > G’’ the formulation behaves like elastic solid. For gel-like materials temperature can deeply affect the behaviour of the two modules and it is possible to observe temperatures values for which G’ < G’, meaning that the formulation behaves like a solution. The crossover point, defined as the temperature where G’ and G’’ assume the same value, is considered the gel point. The gel point is the temperature where the sol/gel or gel/sol transition may be observed from the rheological point of view. In Figure 9 the crossover points for the three tested formulations are reported.

For 20% *w*/*v* PF127 solution the gel point was detected at 23.9 °C, as expected from literature data [40]. For the S-TNH formulation the gel point was 30.4 °C. This data does not seem to be exactly comparable with that obtained from the inversion tube test. However, it must be considered that the measurement obtained by means of the rheometer allows to identify the real gel point, while the gelling temperature observed in the test is determined visually. Therefore, there could be a delay in the appearance of the gel compared to what happens from the molecular point of view in the formulation. The temperature observed in the inversion tube test is in any case compatible with the rheological gel point, being in the temperature range in which the formulation shows an elastic behaviour. The highest value of the crossover point registered for M-TNH (41.8 °C) might suggest that further modification in µE 2 might be necessary to optimize its rheological behaviour with a view to its use in intravitreal administration.

**Figure 10:**
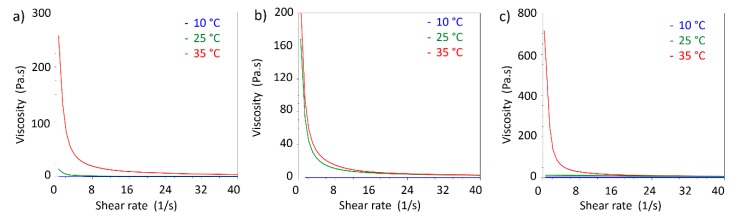
Viscosity curve of: (**a**) 20% *w*/*v* PF127; (**b**) S-TNH; (**c**) M-TNH at different temperatures (10, 25, 35 °C).

In addition, viscosity measurements in shear rate ramp performed at different temperature allowed to compare the viscosity changes with temperature (Figure 10). 

As expected by comparison with viscoelastic measurements, 20% *w*/*v* PF127 shows an increment in viscosity above 25 °C. The same features were observed in S-TNH and M-TNH formulations with the difference that the viscosities at 25 and 35 °C are similar. For all the three formulations, viscosity at 10 °C is nearly independent from shear rate, showing a Newtonian behaviour at that temperature, indicative of the presence of a solution. 

### 4.10. Biocompatibility Studies 

The biocompatibility of the developed nanocomposite systems was tested in separate experiments to evidence the possible different influences on cell viability of the lipid and of the hydrophilic components. Therefore, blank µE1, blank SLN dispersion and 20% *w*/*v* PF127 hydrogel were tested on retinal-pigmented epithelium ARPE-19 cells, employed in this study as a model of ocular epithelial cells. The cells were incubated for 72 h with each sample at four different dilutions (from 1:100 to 1:700) and cell proliferation was determined by SRB assay. 

**Figure 11:**
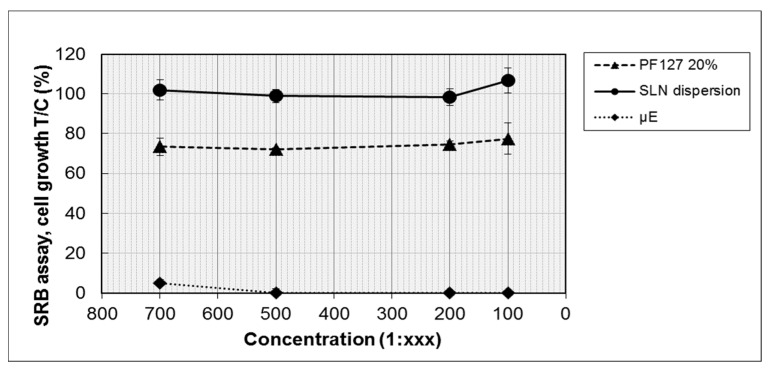
Effect of blank SLN dispersion, µE1 and PF127 20% *w*/*v* hydrogel on ARPE-19 cell proliferation after 72 h of incubation. Cell growth is expressed as the % T/C (mean OD of treated cells/mean OD of control cells × 100). Values are mean ± SD (*n* = 3 wells/condition) of two independent experiments.

As shown in Figure 11, the viability of ARPE-19 cells was affected by µE1, also at the highest dilution tested; only 6% of the viability at the 1:700 dilution was observed. Instead, SLN dispersion and 20% *w*/*v* PF127 hydrogel did not evidence important changes on cell viability, at all the tested dilutions. After 72 h of incubation, SLN dispersion did not show any decrease of cell proliferation, while only a small loss of viability (20–25%), the same for all dilutions, was observed with 20% *w*/*v* PF127 hydrogel. 

Overall, the results revealed that µE1 is very toxic for the cells, whereas neither SLN dispersion nor the polymeric matrix cause a toxic reaction, confirming a certain biocompatibility of the basic ingredients of the SLN nanocomposite system. The toxicity of µE1 might be ascribed to the high content of surfactants and cosurfactant in the formulation: therefore, a further optimization in µE1 composition is required to propose it for intravitreal injection.

### 4.11. Ocular Flow Cell

#### 4.11.1. Simulated Vitreous Viscosity

Viscosity curves on simulated vitreous just prepared and after seven and 14 days from preparation were carried out by a cylinder type rotational viscometer at 35 °C.

Results showed that viscosity curve was unmodified over time, and a non-Newtonian, pseudoplastic behaviour was noted (data not shown). Apparent viscosity, determined in rest conditions, was 6000 mPa·s.

#### 4.11.2. Ocular Flow Cell Dye Clearance 

**Figure 12:**
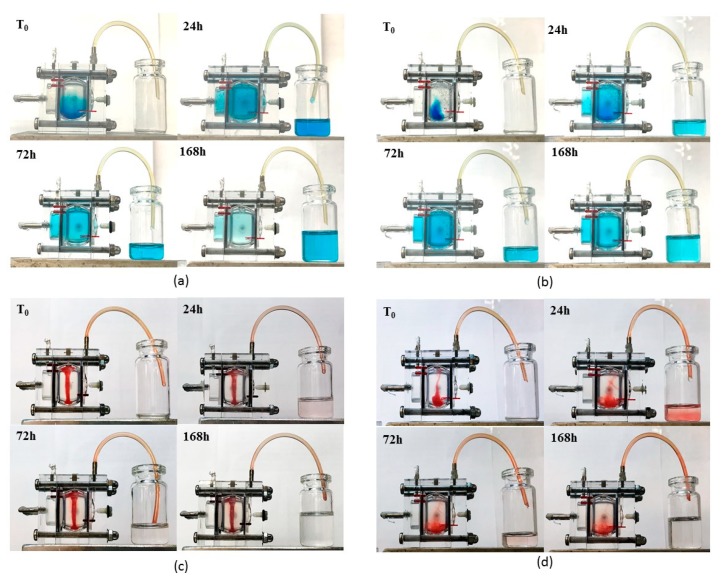
Brilliant Blue ocular cell diffusion and clearance from (**a**) 0.1% *w*/*v* in aqueous solution; (**b**) 0.1% *w*/*v* in 20% *w*/*v* PF127 aqueous solution; Sudan III ocular cell diffusion and clearance from (**c**) 0.1% *w*/*v* in SLN; (**d**) 0.1% *w*/*v* in S-TNH.

In all experiments samples were injected into the posterior cavity. Awwad et al. [26] evidenced the need to operate under an inlet flow to avoid dye concentration in the posterior cavity. These experiments were done to broadly mimic mass transfer in the eye under an aqueous flow and to investigate the different dye transfer and clearance from aqueous solutions and from SLN dispersions. Figure 12 shows the different behaviours at 0, 24, 72, 168 h after injection; a more detailed over time sequence of the experiment is included as supplementary data (Figures S3 and S4). Brilliant Blue was chosen as hydrophilic dye to be dissolved in water and in a 20% *w*/*v* PF127 aqueous solution which converted in a thermoreversible hydrogel around 35 °C. On the other side, the lipophilic Sudan III was introduced in SLN and in S-TNH thanks to its prevailing dissolution in the lipid matrix. Brilliant Blue and Sudan III simulated a high MW hydrophilic and lipophilic drug respectively. The aim of this experiment was to visualize how a hydrophilic and a lipophilic marker diffuses in the eye cell when in solution or SLN-loaded, and to assess whether the temperature-induced thickening of each system might influence dye diffusion and clearance. 

Brilliant Blue in aqueous solution diffused quickly throughout the cell and cleared through the outflow port placed on the anterior chamber: after one week it was nearly completely flown out (Figure 12a). When 20% *w*/*v* PF127 was added, the temperature-induced hydrogel slowed dye diffusion, as the hydrogel itself seemed to be stuck in the bottom of the cell. Only after two days the dye was uniformly distributed throughout the cell and after one week the amount cleared from the outflow port was less than that obtained with the non-thickened aqueous solution (Figure 12b). 

In a different way, when Sudan III-loaded in SLN and in S-TNH was injected, dye diffusion and clearance were very low from both systems. In detail, SLN suspension assumed a column-like shape directed from the inlet port toward the bottom of the vitreous chamber and only a negligible diffusion and clearance of Sudan III was noted in one week (Figure 12c). On the contrary, S-TNH deposed mainly in the bottom of the vitreous chamber and, surprisingly, Sudan III diffused slowly in the receiving medium up to 24 h. Afterwards, Sudan III diffusion and clearance decreased, and in a week, most dye sill remained loaded in S-TNH (Figure 12d). This behaviour, different from that of SLN, probably can be ascribed to the presence of PF127 that allows a more homogeneous nanoparticles dispersion throughout the simulated vitreous and determines a burst release of Sudan III adsorbed onto S-TNH surface. In any case, Sudan III, mimicking a lipophilic drug such as d-CEF, remained mainly located within nanoparticles that, in their turn, diffused very slowly throughout the cell. These studies are still going on to evaluate the possibility to further exploit nanocomposite hydrogels for the intravitreal administration of long-acting drugs for degenerative diseases of the retina: one-month preliminary results are encouraging and will the topic of a following paper.

### 4.12. In Vitro Release Studies

**Figure 13:**
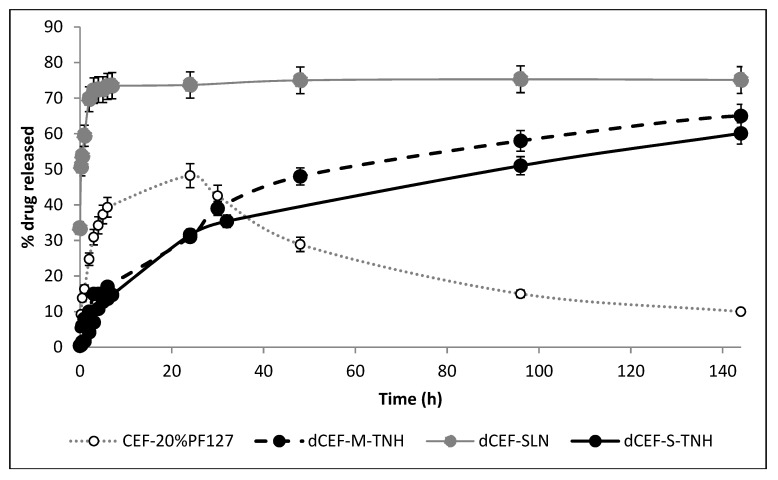
In vitro tube test release of CEF from 20% *w*/*v* PF127 hydrogel (grey line) or dCEF from SLN (grey solid line) and from nanocomposite systems: S-TNH (black solid line) and M-TNH (black dotted line). 15% *v/v* Tween®80 aqueous solution was used as receptor medium. Each bar represents the mean ± SD (*n* = 5). Compared to the control (CEF-20%PF127), during the first 24 h drug release from the nanocomposites was significantly reduced (*p* < 0.05) meanwhile from the 2nd day to the end of the experiment it was significantly increased (*p* < 0.05 to *p* < 0.01). No significant differences were observed between S-TNH and M-TNH, especially during the first 24 h (*p* > 0.05).

In Figure 13 dCEF release profiles from nanocomposite systems, expressed as drug percentage released versus time in a static tube test, are shown and compared with dCEF in SLN dispersion and CEF in 20% *w*/*v* PF127 hydrogel. This experiment does not simulate drug release in physiological conditions because it does not consider the aqueous flow within the eye, thus, it only represents an indirect method to assess drug release profiles from the nanocomposites. As the nanocomposites herein tested are systems consisting in either SLN (S-TNH) or nanodroplets (M-TNH) dispersed in a polymeric network (PF127), it can be reasonably supposed that a two-step process is involved: the release of dCEF from the nanocarrier to the outer hydrogel, and the migration through the polymeric network to the receiving phase. Despite the surfactant receiving phase (15% *v/v* Tween®80) is a non-physiological medium, it has been chosen because the surfactant solution can dissolve dCEF. Considering the first 24 h, it can be noted that when CEF is entrapped as lipophilic dCEF within the lipid internal phase of S-TNH or M-TNH, it is released to the receiving phase significantly slowly than free CEF, confirming drug entrapment within SLN or µE-droplets dispersed in the thermosensitive hydrogel, which can reduce drug release rate. In the case of dCEF-SLN, which were not surrounded by the PF127 network, the presence of Tween®80 in the receiving medium was probably responsible for a partial nanoparticles erosion and of a sudden dissolution of dCEF both adsorbed onto SLN surface and entrapped in SLN, determining a marked burst effect.

Moreover, after 24 h, CEF release rate from its aqueous solution starts to be affected by degradation phenomena, as already reported and discussed above (Figure 3); therefore, the release rate was drastically reduced and the concentration of free CEF gradually decreased to less than 10%. On the contrary, in the esterified form, CEF is more stable, probably due to the shielding effect of the side dodecyl chain. 

Basing on these preliminary data, although being affected by the erosion action of Tween®80 on SLN, the esterification of CEF and the entrapment in a nanocomposite system (especially SLN based hydrogel) contribute to lower the release rate of CEF over a relatively long period of time. 

According to the above mentioned results regarding in vitro biocompatibility, S-TNH was found to be the best of the different formulations developed, and the most suitable one to be proposed for intravitreal administration. For such reasons, it was selected for further release experiment carried out by employing the ocular flow cell described in Section 3.10. 

In these experiments, the release of dCEF from S-TNH was compared with that from SLN, meanwhile the release of CEF from an aqueous solution was compared with that from the hydrogel. 

**Figure 14:**
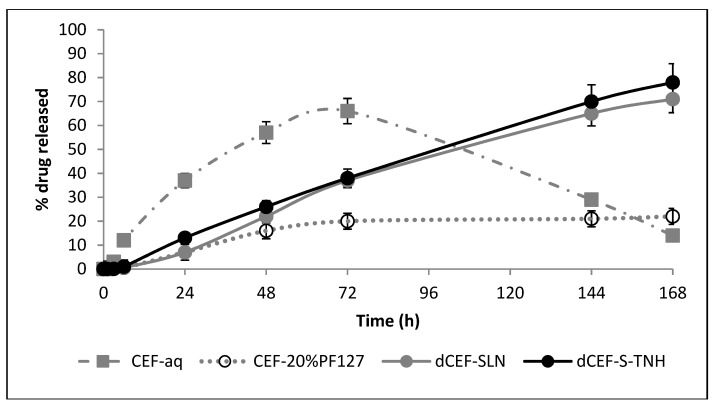
In vitro release test by ocular flow cell of: CEF from aqueous solution (grey dash and dot) and from PF127 20% *w*/*v* hydrogel (grey dotted line) or dCEF from SLN (grey solid line) and from S-TNH (black solid line). Each bar represents the mean ± SD (*n* = 5). Between the two CEF-containing preparations, significance was found from 0 to 72 h (*p* < 0.01) whilst no significant differences were noted for the end-points (*p* > 0.05). Comparing dCEF-SLN and dCEF-S-TNH with CEF-20%PF127, significance was observed from 72 to 168 h (*p* < 0.01). No significant differences were observed between dCEF-SLN and dCEF-S-TNH (*p* > 0.05).

It was noted that in case of CEF, the presence of PF127 significantly slowed the release rate of the drug, being reasonably influenced by the viscosity of the polymer. Additionally, by comparing both SLN samples, a sustained release (up to seven days) of dCEF from both SLN and S-TNH was observed, even if no significant differences were noted between them (Figure 14). We hypothesized that dCEF release from SLN and S-TNH might be mainly controlled by the erosion of the lipid matrix. In any case, the goal to prolong CEF release in the vitreous up to one week was largely achieved.

### 4.13. Microbiological Studies

All the collected samples permitted to isolate colonies of *Staphilococcus epidermidis*. 

The Kirby Bauer test was performed before calculating the MIC values: this two-steps procedure was important both to compare the two methods and as to control for the subsequent MIC tests. The results reported in Table 4 showed that only pure CEF has an effective antibacterial activity at 24 h, 48 h and 72 h. At 96 h, a regrowth of *Staphylococcus epidermidis* on the plates occurred thus it was classified as resistant. No other formulation showed antibacterial activity. The diameter of diffusion, measured equal to zero, shows absence of antibacterial activity.

The results obtained by MIC evaluation (Table 5) showed that only CEF has antibacterial activity. The efficacy of CEF towards *Staphylococcus epidermidis* was calculated at MIC ≤ 0.25 µg/mL at 24, 48, 72 h. Only at 96 h, an increase in the MIC value of 0.4 µg/mL was detected, however this increase is still within the range of CEF sensitivity.

For all other formulations, on the other hand, a MIC value ≥ 0.5 µg/mL was calculated, therefore they did not show antibacterial efficacy. Since there are no references in the international guidelines for MIC values for these formulations, the authors referred to the values reported for CEF according to Clinical and Laboratory Standards Institute (CLSI) guidelines.

## 5. Conclusions

Injectable hydrogels have been recently studied in the treatment of ophthalmic diseases and a number of them are proposed both as surgery sealants, tissue adhesives or vitreous humor substitute and as drug delivery systems [41]. Additionally, SLN have been reported to be an alternative system to emulsions, liposomes, microparticles and their polymeric counterparts for various application routes since the early 1990s, due to their several advantages among which include their biocompatible nature and high drug entrapment efficiency [42]. On the other hand, µE offer several advantages as drug delivery systems as these are thermodynamically stable and, acting as supersolvents of many drugs, they can increase the bioavailability of both lipophilic and hydrophilic molecules.

In this study, SLN and µE-based thermosensitive nanocomposite hydrogels employing PF127, (whose minimal irritation potential is well known [43]) as polymeric matrix were developed and evaluated as intravitreal injectable systems for antibiotic prophylaxis during ocular cataract surgery. Both formulations were able to successfully entrap CEF lipophilic esterified form (dCEF) and were, therefore, studied to develop long-term CEF intraocular delivery systems to prolong its efficacy avoiding topical treatment. This approach combines the intrinsic properties of SLN and µE with the versatility of hydrogels. SLN resulted more performing than µE: the hydrophobic core of SLN can easily accommodate dCEF; moreover, the preparation technique operates at room temperature avoiding degradation phenomena. The resulting dCEF-SLN and dCEF-S-TNH were quite stable over time, since their physico-chemical properties did not change up to one month after preparation, and neither did dCEF in terms of chemical stability. Therefore, it can be assumed that the association with a hydrogel allows to increase the stability of SLN and to slow the release of dCEF for up to seven days, suitable for intraocular antibacterial prophylaxis. The safety of most ingredients used in SLN formulation, such as Cremophor®RH60 and Epikuron®200 is recognized, as they are authorized for pharmaceutical use; moreover, trilaurin is generally considered as a GRAS ingredient [44]. Indeed, neither SLN dispersion nor the polymeric matrix caused a toxic reaction to the ARPE cell line, confirming their biocompatibility; on the contrary, a different result was evidenced for µE-based systems. For this reason, even if the preliminary results of in vitro tube diffusion test were encouraging, dCEF-M-TNH were abandoned and not further studied in eye cell flow studies. Future investigation will be necessary to ameliorate the µE composition in terms of biocompatibility.

Ocular flow cell diffusion and clearance, simulated using both a hydrophilic and a lipophilic dye, together with in vitro CEF release studies, underlined the possibility to exploit SLN-based d-CEF nanocomposite hydrogels to enhance drug permanence in the simulated vitreous and to prolong its release up to one week. 

The results of in vitro dCEF hydrolysis suggested that also *in vivo* a sufficient amount of free CEF might be released from its lipophilic prodrug dCEF, necessary precondition to ensure its antibiotic activity after intravitreal administration: a future step will be the assessment of this hypothesis. 

Considering the results obtained by the microbiological assays, only pure CEF maintained an antimicrobial activity. On the other hand, one of the main objectives of this work was to create a local and long lasting CEF, in order to achieve a reduction of the administered dose. Considering the Kirby Bauer and the MIC results of the present study, further investigations are necessary to better understand how to reach this goal because the clinically-isolated *Staphylococcus intermedius* demonstrated a strong resistance, from the early experimental time points.

Concluding, the preliminary data reported in this paper on SLN-based nanocomposite hydrogels can be considered as a starting point for the future development of carrier systems for different-purpose intraocular administration, such as in age-related degenerative retinal pathologies.

## Figures and Tables

**Table 1:** Percentage of CEF hydrolysis in presence of 5 U of esterase porcine liver.

Incubation Medium	(CEF_f_/dCEF_i_) %.	
	dCEF_i_ ± S.E. (0.3 mg/mL)	dCEF_i_ ± S.E. (0.03 mg/mL)
Triton®X-100 + Tween®80	0.02 ± 0.001	0.05 ± 0.003
Tween®80	0.32 ± 0.02	0.5 ± 0.04

**Table 2:** Composition of dCEF-SLN dispersions (in bold: Composition of µE2).

Component (mg)	dCEF-SLN
Trilaurin	**60**
Ethyl acetate_s_ *	**180**
Epikuron®200	**170**
Cremophor®RH60	**53**
dCEF	**2**
water_s_ *	**700**
Taurocholic acid sodium salt	**10**
PF68 2% *w*/*v* water solution	*q.s.* 5000

* Ethyl acetate and water were mutually presaturated.

**Table 3:** Physicochemical characteristics of blank SLN or dCEF-loaded (dCEF-SLN).

	Mean Diameter (nm) ± S.E.	PDI	*ζ* Potential (mV) ± S.E.	EE% ± S.E.
Blank SLN	271 ± 9.2	0.285	−20 ± 1.6	-
dCEF-SLN	326 ± 9.0	0.231	−18 ± 1.4	78 ± 3.1

**Table 4:** Antimicrobial susceptibility test (Kirby Bauer test).

	Kirby Bauer Agar Diffusion Test (mm)			
Sample	*24 h*	*48 h*	*72 h*	*96 h*
CEF	32 S	31 S	31 S	(m.o regrowth) R
dCEF	0 R	0 R	0 R	0 R
blank SLN	0 R	0 R	0 R	0 R
blank S-TNH	0 R	0 R	0 R	0 R
dCEF-SLN	0 R	0 R	0 R	0 R
dCEF-S-TNH	0 R	0 R	0 R	0 R

**Table 5:** MIC test results.

	MIC (µg/mL)			
Sample	*24 h*	*48 h*	*72 h*	*96 h*
CEF	≤0.25 S	≤0.25 S	≤0.25 S	0.4 S
dCEF	≥0.5 R	≥0.5 R	≥0.5 R	≥0.5 R
blank SLN	≥0.5 R	≥0.5 R	≥0.5 R	≥0.5 R
blank S-TNH	≥0.5 R	≥0.5 R	≥0.5 R	≥0.5 R
dCEF-SLN	≥0.5 R	≥0.5 R	≥0.5 R	≥0.5 R
dCEF-S-TNH	≥0.5 R	≥0.5 R	≥0.5 R	≥0.5 R

## Supplementary Materials

The following are available online at https://www.mdpi.com/2079-4991/9/10/1461/s1, Figure S1: 1H-NMR spectrum of dCEF in CDCl3, Figure S2: 13C-NMR spectrum of dCEF in CDCl3, Figure S3: Brilliant Blue ocular cell diffusion and clearance from: 0.1% *w*/*v* in aqueous solution (left); 0.1% *w*/*v* in 20% *w*/*v* PF127 aqueous solution (right), Figure S4: Sudan III ocular cell diffusion and clearance from: 0.1% *w*/*v* in SLN (left); 0.1% *w*/*v* in S-TNH (right).
