# Supplementary material for: Thermosensitive Nanocomposite Hydrogels for Intravitreal Delivery of Cefuroxime
Source: Nanomaterials (Basel). 2019 Oct 15;9(10):1461. doi: 10.3390/nano9101461 (PMC6835325; doi:10.3390/nano9101461)
Supplement: Supplementary file 1 [file nanomaterials-09-01461-s001.pdf]

SUPPLEMENTARY FIGURE CAPTIONS

Thermosensitive Nanocomposite Hydrogels for Intravitreal Delivery of Cefuroxime

Simona Sapino [1,*,†] Elena Peira [1,†], Daniela Chirio [1,*], Giulia Chindamo [1], Stefano Guglielmo [1], Simonetta Oliaro-Bosso [1], Raffaella Barbero [2], Cristina Vercelli [3], Giovanni Re [3], Valentina Brunella [4], Chiara Riedo [4], Antonio Maria Fea [5] and Marina Gallarate [1]

[1] Department of Drug Science and Technology, University of Turin, Turin 10125, Italy; elena.peira@unito.it (E.P.); giulia.chindamo@unito.it (G.C.); stefano.guglielmo@unito.it (S.G.); simona.oliaro@unito.it (S.O.); marina.gallarate@unito.it (M.G.)

[2] SC of Serology, Istituto Zooprofilattico Sperimentale Piemonte Liguria e Valle d'Aosta, Turin 10154, Italy; raffaella.barbero@izsto.it (R.B.)

[3] Department of Veterinary Sciences of Turin, University of Turin, Turin 10095, Italy; cristina.vercelli@unito.it (C.V.); giovanni.re@unito.it (G.R.)

[4] Department of Chemistry, University of Turin, Turin 10125, Italy; valentina.brunella@unito.it (V.B.); chiara.riedo@unito.it (C.R.)

[5] Department of Surgical Sciences, University of Turin, Turin 10126, Italy; antoniomaria.fea@unito.it (A.M.F.)

* Correspondence: simona.sapino@unito.it (S.S.); daniela.chirio@unito.it (D.C.); Tel.: +39-011-6706800 (S.S.)

† These authors contributed equally to this work.

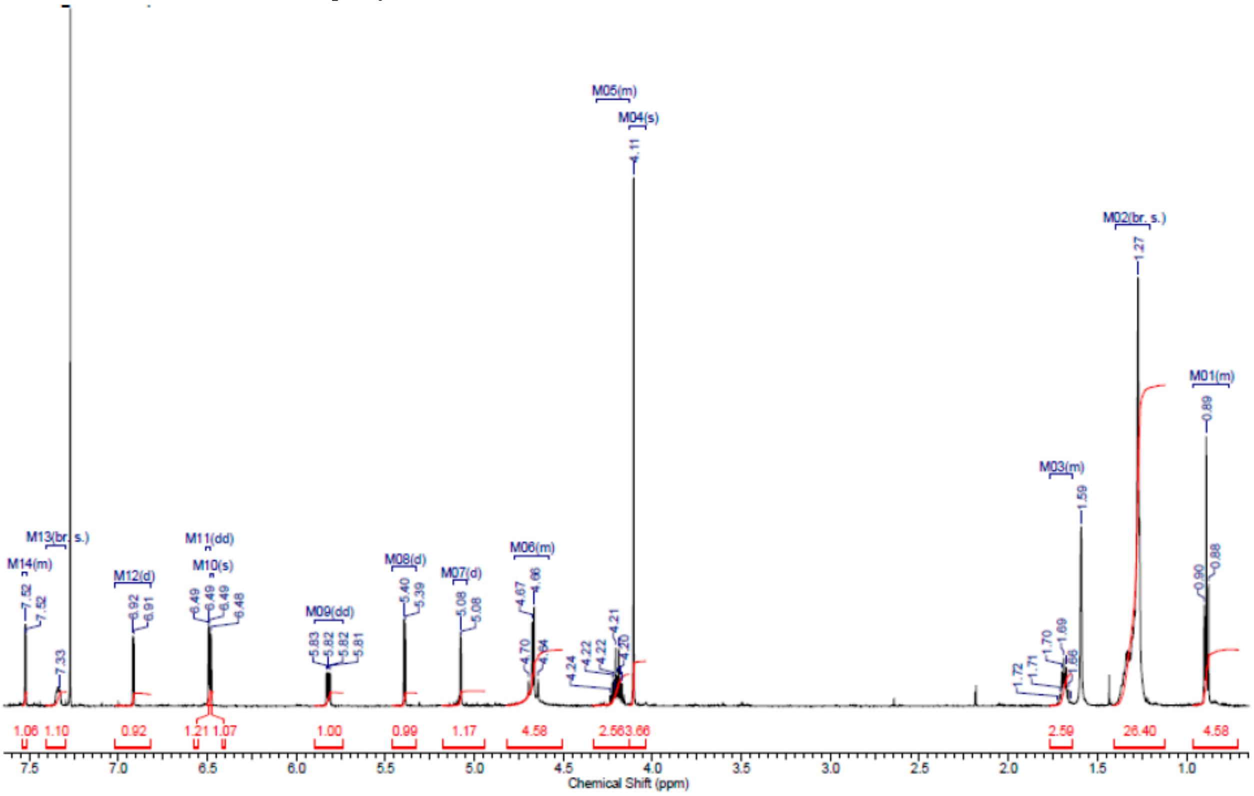

Figure S1. ^{1}H-NMR spectrum of dCEF in CDCl3.

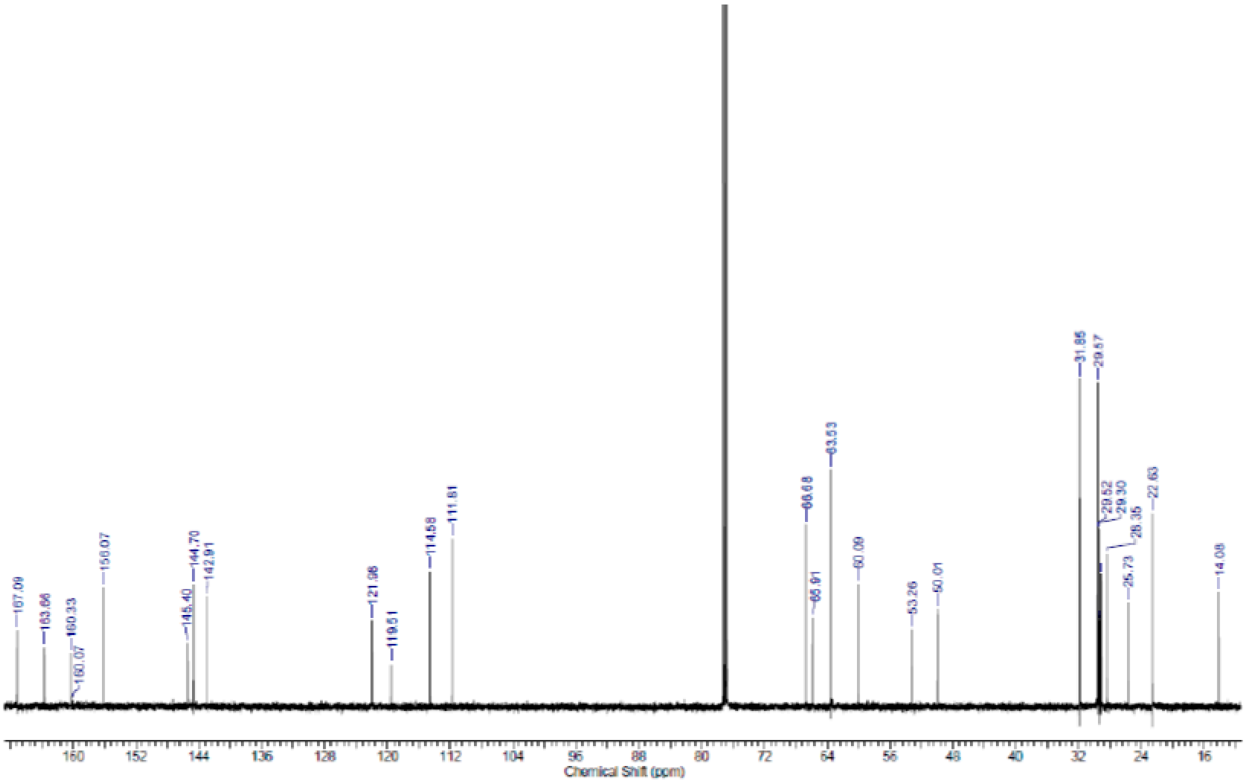

Figure S2. ^{13}C-NMR spectrum of dCEF in CDCl3.

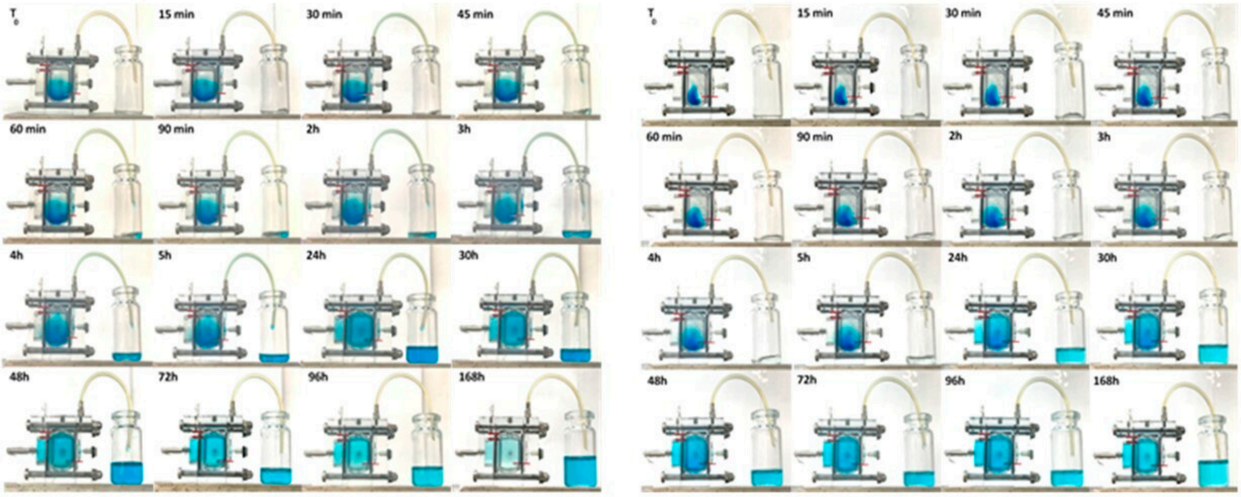

Figure S3. Brilliant Blue ocular cell diffusion and clearance from: 0.1% w/v in aqueous solution (left); 0.1% w/v in 20% w/v PF127 aqueous solution (right).

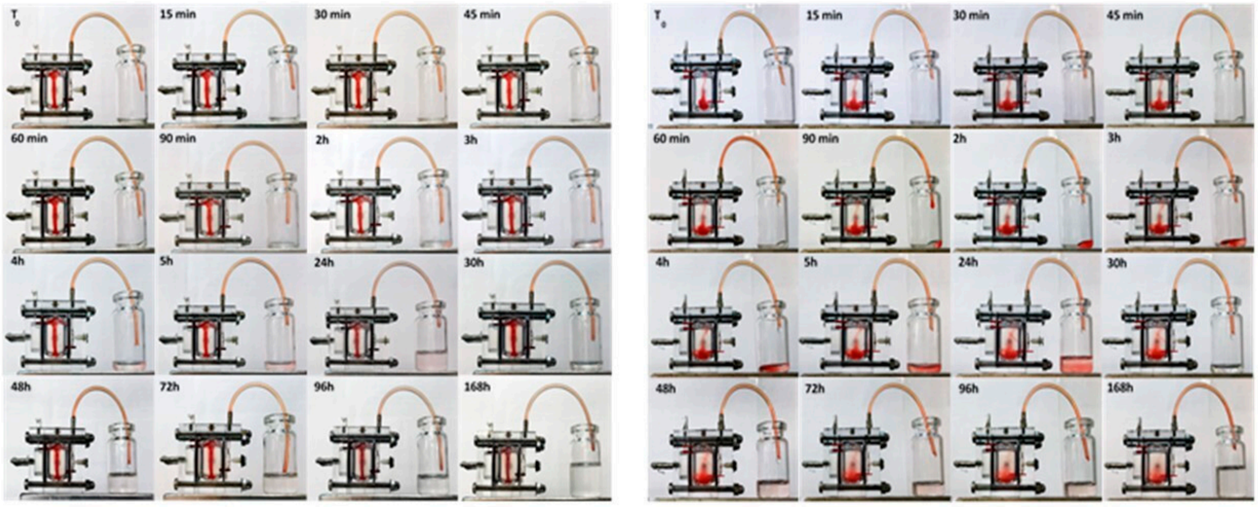

Figure S4. Sudan III ocular cell diffusion and clearance from: 0.1% w/v in SLN (left); 0.1% w/v in S-TNH (right).
